# Supplementary material for: Nox4 expression in osteo-progenitors controls bone development in mice during early life
Source: Commun Biol. 2022 Jun 14;5:583. doi: 10.1038/s42003-022-03544-0 (PMC9198054; doi:10.1038/s42003-022-03544-0)
Supplement: Supplementary file 2 — Supplementary Information [file 42003_2022_3544_MOESM2_ESM.pdf]

Supplementary Information

Nox4 expression in osteo-progenitors controls bone development in mice during early life

Supplementary Figure 1

Supplementary Figure 2

Supplementary Tables 1-9

Supplementary Results: bone marrow RNA sequencing

Supplementary Figures

Suppemental Fig. 1

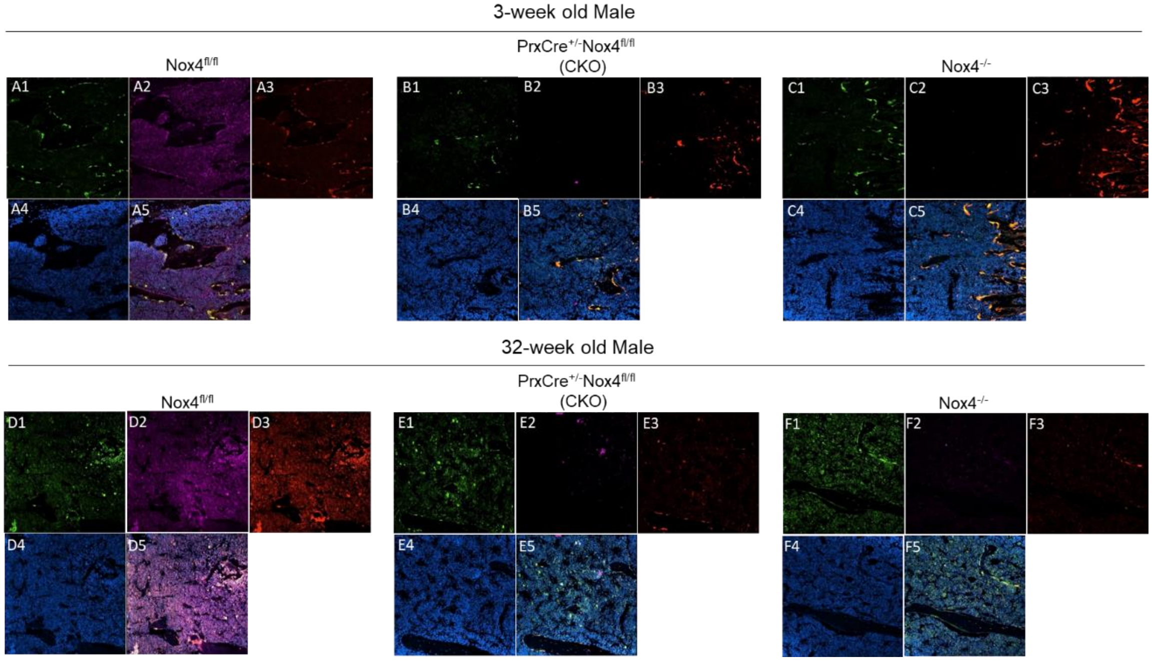

Supplemental Fig. 1. Co-immunostainings on 3-week old and 32-week old male femur histological sections for Prx1 (green, A1, B1, C1, D1, E1, F1)
Nox4 (purple, A2, B2, C2, D2, E2, F2) and Cathepsin k (orange, A3, B3, C3, D3, E3, F3) for osteoblastic and osteoclastic. A4-F4, DAPI; A5-F5, color merged.

Supplementary Fig. 2

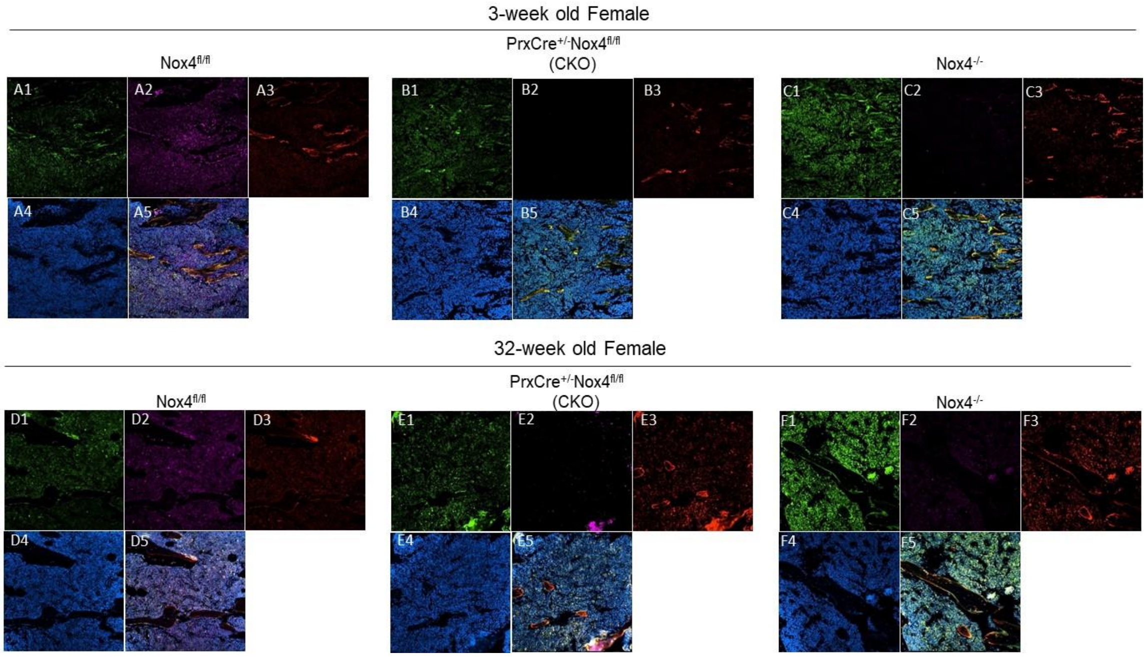

Supplemental Fig. 2. Co-immunostainings on 3-week old and 32-week old female femur histological sections for Prx1 (green, A1, B1, C1, D1, E1, F1)
Nox4 (purple, A2, B2, C2, D2, E2, F2) and Cathepsin k (orange, A3, B3, C3, D3, E3, F3) for osteoblastic and osteoclastic. A4-F4, DAPI; A5-F5, color merged.

Supplementary Table 1. Tibia bone micro-CT trabecular bone parameters in 32-week old male and female wild type and PreCre^{+}/-Nox4$^{fl/fl}$ (CKO) and Nox4$^{-/-}$ mice

			Males			P Value			Females			P Value		
						Nox4$^{fl/fl}$	CKO	Nox4$^{fl/fl}$				Nox4$^{fl/fl}$	CKO	Nox4$^{fl/fl}$
				PrxCre$^{+/-}$Nox4$^{fl/fl}$		vs	vs	vs		PrxCre$^{+/-}$Nox4$^{fl/fl}$		vs	vs	vs
	Abbreviation	Units	Nox4$^{fl/fl}$	CKO	Nox4$^{-/-}$	CKO	Nox4$^{-/-}$	Nox4$^{-/-}$	Nox4$^{fl/fl}$	CKO	Nox4$^{-/-}$	CKO	Nox4$^{-/-}$	Nox4$^{-/-}$
Tissue volume	TV	mm^3	2.73 ± 0.03	2.59 ± 0.13	2.54 ± 0.18	0.007	0.284	0.007	2.46 ± 0.2	2.38 ± 0.2	2.11 ± 0.13	0.194	0.022	0.004
Bone volume	BV	mm^3	0.15 ± 0.01	0.18 ± 0.04	0.17 ± 0.05	0.035	0.365	0.112	0.06 ± 0.01	0.06 ± 0.01	0.05 ± 0.01	0.297	0.114	0.048
Percent bone volume	BV/TV	%	5.51 ± 0.39	6.49 ± 0.55	6.88 ± 1.77	0.001	0.294	0.034	2.54 ± 0.52	2.5 ± 0.48	2.41 ± 0.65	0.437	0.394	0.344
Tissue surface	TS	mm^2	14.99 ± 0.16	14.43 ± 0.64	14.42 ± 0.68	0.022	0.491	0.026	13.86 ± 0.86	13.61 ± 0.78	12.77 ± 0.65	0.276	0.051	0.021
Bone surface	BS	mm^2	12.38 ± 0.8	13.25 ± 2.4	12.57 ± 2.08	0.190	0.290	0.414	5.13 ± 1.02	5.01 ± 0.77	3.95 ± 0.99	0.403	0.039	0.036
Intersection surface	i.S	mm^2	0.6 ± 0.05	0.72 ± 0.14	0.76 ± 0.26	0.024	0.355	0.064	0.31 ± 0.07	0.29 ± 0.07	0.25 ± 0.05	0.272	0.225	0.097
Bone surface / volume ratio	BS/BV	1/mm	82.54 ± 6.31	73.11 ± 4.17	74.13 ± 9.94	0.003	0.403	0.042	82.29 ± 7.18	85.18 ± 6.2	77.96 ± 4.66	0.201	0.038	0.146
Bone surface density	BS/TV	1/mm	4.52 ± 0.26	5.06 ± 0.64	4.96 ± 0.74	0.031	0.390	0.086	2.1 ± 0.48	2.11 ± 0.29	1.89 ± 0.58	0.470	0.211	0.258
Trabecular pattern factor	Tb.Pf	1/mm	34.55 ± 3.83	30.69 ± 4.97	30.47 ± 5.76	0.065	0.471	0.073	34.9 ± 4.18	36.39 ± 3.72	33.48 ± 1.87	0.230	0.092	0.268
Centroid (x)	Crd.X	mm	1.97 ± 0.3	1.92 ± 0.26	1.75 ± 0.28	0.360	0.132	0.087	1.96 ± 0.17	1.98 ± 0.14	1.86 ± 0.18	0.416	0.120	0.160
Centroid (y)	Crd.Y	mm	1.73 ± 0.17	1.67 ± 0.17	1.71 ± 0.34	0.271	0.384	0.464	1.67 ± 0.25	1.76 ± 0.26	1.96 ± 0.12	0.238	0.088	0.023
Centroid (z)	Crd.Z	mm	17.72 ± 0.43	17.65 ± 1.32	17.18 ± 1.43	0.448	0.270	0.182	17.54 ± 1.01	17.15 ± 0.66	17.71 ± 0.98	0.192	0.142	0.392
Structure model index	SMI		2.5 ± 0.11	2.51 ± 0.3	2.45 ± 0.22	0.495	0.358	0.294	2.54 ± 0.14	2.56 ± 0.12	2.58 ± 0.18	0.376	0.409	0.322
Trabecular thickness	Tb.Th	mm	0.05 ± 0	0.05 ± 0	0.05 ± 0.01	0.001	0.327	0.025	0.05 ± 0	0.05 ± 0	0.05 ± 0.01	0.322	0.096	0.088
Trabecular number	Tb.N	1/mm	1.16 ± 0.04	1.21 ± 0.16	1.29 ± 0.25	0.205	0.228	0.085	0.52 ± 0.12	0.52 ± 0.1	0.47 ± 0.17	0.489	0.290	0.279
Trabecular separation	Tb.Sp	mm	0.28 ± 0.02	0.27 ± 0.01	0.28 ± 0.02	0.071	0.247	0.261	0.42 ± 0.06	0.41 ± 0.02	0.43 ± 0.04	0.322	0.129	0.377
Degree of anisotropy	DA		2.32 ± 0.07	2.47 ± 0.2	2.47 ± 0.12	0.036	0.416	0.165	2.38 ± 0.39	2.29 ± 0.29	2.23 ± 0.13	0.136	0.213	0.236
Eigenvalue 1			4.37 ± 1.37	3.85 ± 0.77	3.49 ± 1.44	0.202	0.286	0.134	4.58 ± 1.19	4.96 ± 1.28	2.7 ± 0.55	0.273	0.005	0.006
Eigenvalue 2			8.38 ± 2.55	7.48 ± 1.72	6.49 ± 2.84	0.228	0.221	0.107	7.37 ± 1.87	7.71 ± 2.61	4.44 ± 0.85	0.378	0.020	0.006
Eigenvalue 3			10.18 ± 3.27	9.5 ± 1.77	8.58 ± 3.52	0.319	0.273	0.198	10.56 ± 1.8	11.15 ± 2.35	6.24 ± 0.87	0.284	0.002	0.000

Supplemental Table 1. Micro-CT trabecular bone parameters of tibia of 32-week old male and female Nox4$^{fl/fl}$ control, and PrxCre$^{+/-}$ Nox4$^{fl/fl}$ CKO and Nox4$^{-/-}$ mice. There were 6-9 mice per group. Data was expressed as mean ± SD, p value was analyzed by 1-way ANOVA with posthoctests, CKO versus f/f control or Nox4$^{-/-}$.

Supplementary Table 2. Femur bone micro-CT cortical bone parameters in 3-week old male and female wild type and PreCre^{+}/-Nox4$^{fl/fl}$ (CKO) and Nox4$^{-/-}$ mice

			Males						Females					
						Nox4$^{fl/fl}$	CKO	Nox4$^{fl/fl}$				Nox4$^{fl/fl}$	CKO	Nox4$^{fl/fl}$
				PrxCre$^{+/-}$Nox4$^{fl/fl}$		vs	vs	vs		PrxCre$^{+/-}$Nox4$^{fl/fl}$		vs	vs	vs
	Abbreviation	Units	Nox4$^{fl/fl}$	CKO	Nox4$^{-/-}$	CKO	Nox4$^{-/-}$	Nox4$^{-/-}$	Nox4$^{fl/fl}$	CKO	Nox4$^{-/-}$	CKO	Nox4$^{-/-}$	Nox4$^{-/-}$
Tissue volume	TV	mm^3	1.39±0.15	1.24±0.07	1.36±0.07	0.028	0.015	0.329	1.3±0.2	1.1±0.08	1.3±0.1	0.007	0.036	0.173
Bone volume	BV	mm^3	0.34±0.05	0.29±0.02	0.33±0.03	0.030	0.020	0.303	0.32±0.05	0.25±0.02	0.35±0.04	0.011	0.000	0.120
Percent bone volume	BV/TV	%	24.6±1.1	23.5±1.0	23.0±0.5	0.050	0.188	0.006	24.8±1.8	22.7±1.2	23.6±1.3	0.024	0.123	0.094
Tissue surface	TS	mm^2	8.0±0.6	7.5±0.8	7.5±0.4	0.158	0.428	0.071	8.2±1.5	6.97±1.0	7.7±0.4	0.067	0.044	0.217
Peripheral tissue surface	TS(per)	mm^2	4.5±0.23	4.3±0.13	4.6±0.2	0.027	0.012	0.300	4.66±0.5	4.1±0.2	4.6±0.3	0.020	0.002	0.412
Bone surface	BS	mm^2	10.0±0.7	9.3±0.4	9.9±0.4	0.040	0.029	0.421	9.6±0.6	8.7±0.4	9.8±0.5	0.009	0.001	0.220
Peripheral bone surface	BS(per)	mm^2	8.5±0.5	8.0±0.29	8.4±0.24	0.036	0.020	0.380	8.2±0.5	7.6±0.3	8.4±0.4	0.013	0.001	0.253
Bone surface / volume ratio	BS/BV	1/mm	29.7±2.7	32.1±1.3	30.2±1.4	0.047	0.027	0.342	30.6±3.7	34.5±2.0	28.4±2.2	0.028	0.000	0.100
Mean total crossectional tissue area	T.Ar	mm^2	1.4±0.1	1.2±0.07	1.3±0.07	0.028	0.015	0.329	1.3±0.16	1.1±0.08	1.3±0.1	0.007	0.005	0.442
Mean total crossectional tissue perimeter	T.Pm	mm	4.5±0.2	4.2±0.1	4.6±0.23	0.027	0.012	0.300	4.5±0.3	4.0±0.2	4.5±0.3	0.007	0.002	0.324
Mean total crossectional bone area	B.Ar	mm^2	0.3±0.05	0.29±0.02	0.32±0.03	0.030	0.020	0.303	0.32±0.05	0.25±0.02	0.34±0.04	0.011	0.000	0.120
Mean total crossectional bone perimeter	B.Pm	mm	8.4±0.5	7.9±0.28	8.3±0.24	0.036	0.020	0.380	8.15±0.5	7.5±0.3	8.3±0.4	0.013	0.001	0.253
Average object area per slice	Av.Obj.Ar	mm^2	0.33±0.05	0.29±0.02	0.32±0.03	0.030	0.020	0.303	0.32±0.05	0.25±0.02	0.34±0.04	0.011	0.000	0.120
Average object area-equivalent circle diameter per slice	Av.Obj.ECDa	mm	0.65±0.05	0.60±0.02	0.64±0.03	0.032	0.020	0.317	0.63±0.05	0.56±0.03	0.66±0.04	0.011	0.000	0.119
Average moment of inertia (x)	Av.MMI(x)	mm^4	0.055±0.01	0.041±0.004	0.054±0.01	0.013	0.013	0.415	0.048±0.01	0.033±0.004	0.055±0.01	0.013	0.000	0.091
Average moment of inertia (y)	Av.MMI(y)	mm^4	0.076±0.02	0.058±0.007	0.07±0.01	0.045	0.030	0.295	0.07±0.02	0.045±0.008	0.076±0.02	0.005	0.002	0.240
Mean polar moment of inertia	MMI(polar)	mm^4	0.13±0.03	0.099±0.01	0.12±0.02	0.027	0.011	0.331	0.12±0.03	0.078±0.01	0.13±0.03	0.007	0.001	0.163
Average principal moment of inertia (max)	Av.MMI(max)	mm^4	0.078±0.02	0.058±0.007	0.074±0.01	0.037	0.015	0.357	0.07±0.02	0.046±0.008	0.079±0.02	0.005	0.001	0.202
Average principal moment of inertia (min)	Av.MMI(min)	mm^4	0.053±0.01	0.04±0.004	0.050±0.006	0.017	0.011	0.288	0.046±0.01	0.033±0.004	0.053±0.01	0.014	0.001	0.120
Mean eccentricity	Ecc		0.55±0.06	0.55±0.05	0.56±0.04	0.472	0.321	0.292	0.59±0.04	0.52±0.05	0.56±0.04	0.012	0.056	0.154
Crossectional thickness	Cs.Th	mm	0.08±0.008	0.07±0.003	0.077±0.005	0.039	0.049	0.293	0.077±0.009	0.07±0.005	0.08±0.007	0.021	0.001	0.105
Trabecular thickness (plate model)	Tb.Th(pl)	mm	0.068±0.006	0.062±0.002	0.066±0.003	0.039	0.046	0.254	0.066±0.008	0.058±0.003	0.07±0.006	0.025	0.001	0.104
Trabecular separation (plate model)	Tb.Sp(pl)	mm	0.21±0.007	0.20±0.005	0.21±0.007	0.038	0.114	0.319	0.2±0.02	0.2±0.006	0.2±0.02	0.466	0.286	0.281
Trabecular number (plate model)	Tb.N(pl)	1/mm	3.6±0.15	3.7±0.06	3.6±0.1	0.018	0.030	0.262	3.7±0.22	3.9±0.1	3.6±0.3	0.022	0.054	0.477
Trabecular diameter (rod model)	Tb.Dm(rd)	mm	0.14±0.01	0.12±0.005	0.13±0.007	0.039	0.046	0.254	0.13±0.02	0.12±0.007	0.14±0.01	0.025	0.001	0.104
Trabecular separation (rod model)	Tb.Sp(rd)	mm	0.11±0.004	0.10±0.002	0.10±0.003	0.023	0.053	0.278	0.1±0.006	0.1±0.003	0.1±0.009	0.177	0.118	0.273
Trabecular number (rod model)	Tb.N(rd)	1/mm	4.1±0.26	4.4±0.1	4.2±0.1	0.029	0.019	0.271	4.1±0.5	4.6±0.2	4.0±0.3	0.024	0.001	0.375
Closed porosity (percent)	Po(cl)	%	75.4±1.1	76.3±1.2	74.8±2.4	0.101	0.120	0.290	74.7±3.5	76.9±1.3	72.5±4.6	0.098	0.034	0.164
Centroid (x)	Crd.X	mm	1.4±0.12	1.2±0.2	1.2±0.3	0.163	0.492	0.179	1.3±0.2	1.2±0.1	1.1±0.1	0.083	0.400	0.080
Centroid (y)	Crd.Y	mm	1.21±0.13	1.27±0.15	1.15±0.2	0.216	0.162	0.288	1.15±0.2	1.1±0.1	1.1±0.1	0.287	0.148	0.344
Centroid (z)	Crd.Z	mm	7.25±0.04	7.6±0.6	7.45±0.3	0.103	0.312	0.164	7.3±0.5	6.9±0.5	7.3±0.6	0.112	0.114	0.394
Mean fractal dimension	FD		1.17±0.04	1.16±0.02	1.18±0.04	0.434	0.221	0.319	1.2±0.02	1.2±0.02	1.1±0.02	0.324	0.048	0.083
Total intersection surface	i.S	mm^2	4.52±0.23	4.3±0.13	4.5±0.2	0.029	0.011	0.401	4.8±0.9	4.0±0.2	4.6±0.3	0.045	0.002	0.308

Supplemental Table 2. Micro-CT cortical bone parameters of femur of 3-week old male and female Nox4$^{fl/fl}$ control, and PrxCre$^{+/-}$ Nox4$^{fl/fl}$ CKO and Nox4$^{-/-}$ mice. There were 6-9 mice per group. Data was expressed as mean ± SD, p value was analyzed by 1-way ANOVA with posthoctests, CKO versus f/f control or Nox4$^{-/-}$.

Supplemental Table 3. Femur bone micro-CT cortical bone parameters in 32-week old male and female wild type and PreCre^{+}/-Nox4$^{fl/fl}$ (CKO) and Nox4$^{-/-}$ mice

			Males						Females					
	Abbreviation	Units	Nox4$^{fl/fl}$	PrxCre$^{+/-}$Nox4$^{fl/fl}$ CKO	Nox4$^{-/-}$	Nox4$^{fl/fl}$ vs CKO	CKO vs Nox4$^{-/-}$	Nox4$^{fl/fl}$ vs Nox4$^{-/-}$	Nox4$^{fl/fl}$	PrxCre$^{+/-}$Nox4$^{fl/fl}$ CKO	Nox4$^{-/-}$	Nox4$^{fl/fl}$ vs CKO	CKO vs Nox4$^{-/-}$	Nox4$^{fl/fl}$ vs Nox4$^{-/-}$
Tissue volume	TV	mm^3	2.1±0.2	2.1±0.1	2.0±0.3	0.410	0.330	0.261	1.7±0.2	1.6±0.1	1.6±0.1	0.132	0.232	0.294
Bone volume	BV	mm^3	0.95±0.05	0.96±0.07	0.95±0.06	0.365	0.401	0.469	0.9±0.09	0.8±0.08	0.9±0.05	0.285	0.222	0.447
Percent bone volume	BV/TV	%	44.7±2.8	45.6±1.1	46.7±2.7	0.260	0.216	0.097	53.0±2.4	54.5±2.5	54.6±1.6	0.118	0.454	0.078
Tissue surface	TS	mm^2	10.7±0.8	11.0±0.9	10.6±0.9	0.253	0.214	0.413	9.4±1.3	8.8±0.5	9.4±0.9	0.115	0.054	0.498
Peripheral tissue surface	TS(per)	mm^2	5.9±0.25	6.2±0.5	5.9±0.3	0.103	0.165	0.410	5.3±0.5	5.1±0.2	5.5±0.5	0.118	0.030	0.241
Bone surface	BS	mm^2	14.4±0.9	15.4±1.6	14.5±1.6	0.100	0.178	0.482	11.8±1.0	11.4±0.8	11.8±0.7	0.185	0.199	0.448
Peripheral bone surface	BS(per)	mm^2	11.0±0.6	11.6±1.1	11.1±1.1	0.122	0.209	0.475	9.0±0.7	8.7±0.5	9.0±0.5	0.136	0.139	0.445
Bone surface / volume ratio	BS/BV	1/mm	15.2±0.6	16.0±0.8	15.1±0.7	0.038	0.047	0.447	12.9±0.4	12.7±0.5	12.7±0.5	0.300	0.465	0.274
Mean total crossectional tissue area	T.Ar	mm^2	2.1±0.2	2.0±0.1	2.0±0.3	0.410	0.330	0.261	1.7±0.2	1.6±0.1	1.6±0.1	0.132	0.232	0.294
Mean total crossectional tissue perimeter	T.Pm	mm	5.8±0.3	6.1±0.5	5.9±0.3	0.103	0.165	0.410	5.2±0.5	5.0±0.2	5.4±0.5	0.118	0.030	0.241
Mean total crossectional bone area	B.Ar	mm^2	0.9±0.05	0.9±0.07	0.9±0.06	0.365	0.401	0.469	0.9±0.09	0.8±0.08	0.9±0.05	0.285	0.222	0.447
Mean total crossectional bone perimeter	B.Pm	mm	10.9±0.6	11.5±1.1	11.0±1.1	0.122	0.209	0.475	8.9±0.7	8.6±0.5	8.9±0.5	0.136	0.139	0.445
Average object area per slice	Av.Obj.Ar	mm^2	0.9±0.05	0.9±0.07	0.9±0.06	0.365	0.401	0.469	0.9±0.09	0.8±0.08	0.9±0.05	0.285	0.222	0.447
Average object area-equivalent circle diameter per slice	Av.Obj.ECDa	mm	1.0±0.03	1.1±0.04	1.0±0.04	0.370	0.402	0.473	1.0±0.05	1.0±0.05	1.0±0.03	0.285	0.212	0.436
Average moment of inertia (x)	Av.MMI(x)	mm^4	0.15±0.03	0.13±0.02	0.14±0.03	0.175	0.222	0.398	0.12±0.02	0.11±0.01	0.11±0.01	0.067	0.154	0.236
Average moment of inertia (y)	Av.MMI(y)	mm^4	0.4±0.06	0.4±0.07	0.4±0.09	0.322	0.411	0.430	0.29±0.09	0.26±0.06	0.29±0.05	0.204	0.134	0.495
Mean polar moment of inertia	MMI(polar)	mm^4	0.56±0.09	0.56±0.08	0.56±0.1	0.488	0.494	0.481	0.42±0.1	0.37±0.07	0.41±0.06	0.149	0.132	0.444
Average principal moment of inertia (max)	Av.MMI(max)	mm^4	0.41±0.06	0.42±0.07	0.44±0.08	0.379	0.412	0.280	0.30±0.08	0.27±0.06	0.31±0.05	0.152	0.086	0.462
Average principal moment of inertia (min)	Av.MMI(min)	mm^4	0.14±0.04	0.13±0.02	0.12±0.03	0.298	0.256	0.143	0.11±0.02	0.10±0.02	0.10±0.01	0.155	0.453	0.125
Mean eccentricity	Ecc		0.8±0.04	0.8±002	0.8±0.02	0.217	0.053	0.021	0.78±0.03	0.77±0.03	0.8±0.03	0.287	0.016	0.054
Crossectional thickness	Cs.Th	mm	0.17±0.006	0.16±0.006	0.17±0.007	0.045	0.058	0.447	0.2±0.008	0.2±0.01	0.2±0.01	0.272	0.477	0.293
Trabecular thickness (plate model)	Tb.Th(pl)	mm	0.13±0.006	0.12±0.006	0.13±0.006	0.040	0.046	0.439	0.15±0.005	0.15±0.006	0.15±0.01	0.287	0.464	0.255
Trabecular separation (plate model)	Tb.Sp(pl)	mm	0.16±0.02	0.14±0.01	0.15±0.01	0.103	0.429	0.085	0.13±0.01	0.13±0.008	0.13±0.01	0.078	0.442	0.053
Trabecular number (plate model)	Tb.N(pl)	1/mm	3.4±0.3	3.6±0.3	3.5±0.1	0.065	0.166	0.131	3.4±0.09	3.4±0.06	3.4±0.09	0.070	0.460	0.090
Trabecular diameter (rod model)	Tb.Dm(rd)	mm	0.26±0.01	0.25±0.01	0.26±0.01	0.040	0.046	0.439	0.31±0.01	0.31±0.01	0.31±0.01	0.287	0.464	0.255
Trabecular separation (rod model)	Tb.Sp(rd)	mm	0.08±0.01	0.07±0.008	0.07±0.007	0.113	0.493	0.082	0.067±0.007	0.062±0.006	0.062±0.004	0.087	0.450	0.057
Trabecular number (rod model)	Tb.N(rd)	1/mm	2.8±0.2	3.0±0.2	2.9±0.09	0.043	0.069	0.233	2.6±0.06	2.6±0.06	2.6±0.08	0.342	0.492	0.384
Closed porosity (percent)	Po(cl)	%	55.1±2.8	53.9±0.7	53.1±2.7	0.192	0.270	0.095	46.6±2.4	45.3±2.6	45.1±1.6	0.143	0.421	0.081
Centroid (x)	Crd.X	mm	1.7±0.1	1.6±0.09	1.6±0.1	0.175	0.487	0.173	1.5±0.2	1.4±0.1	1.5±0.1	0.030	0.010	0.435
Centroid (y)	Crd.Y	mm	1.4±0.2	1.3±0.09	1.3±0.2	0.183	0.237	0.438	1.2±0.07	1.2±0.09	1.3±0.1	0.283	0.190	0.260
Centroid (z)	Crd.Z	mm	13.1±0.7	13.0±0.5	13.6±0.5	0.436	0.034	0.047	13.0±0.6	12.6±0.8	13.1±0.6	0.121	0.133	0.460
Mean fractal dimension	FD		1.2±0.02	1.2±0.02	1.2±0.01	0.147	0.230	0.304	1.2±0.02	1.2±0.02	1.2±0.02	0.014	0.009	0.204
Total intersection surface	i.S	mm^2	5.9±0.3	5.9±0.3	5.9±0.3	0.432	0.494	0.428	5.3±0.4	5.1±0.2	5.3±0.3	0.135	0.066	0.455

Supplemental Table 3. Micro-CT cortical bone parameters of femur of 32-week old male and female Nox4$^{fl/fl}$ control, PrxCre$^{+/-}$ Nox4$^{fl/fl}$ CKO and Nox4$^{-/-}$ mice. There were 6 – 9 mice per group. Data was expressed as mean ± SD, p value was analyzed by 1-way ANOVA with posthoctests, CKO versus f/f control or Nox4-/-.

Supplemental Table 4. **Femur** bone micro-CT trabecular bone parameters in 6-week old male and female wild type and PreCre^{+}/-Nox4$^{fl/fl}$ (CKO) and Nox4$^{-/-}$ mice

			Males						Females					
						Nox4$^{fl/fl}$	CKO	Nox4$^{fl/fl}$				Nox4$^{fl/fl}$	CKO	Nox4$^{fl/fl}$
				PrxCre$^{+/-}$Nox4$^{fl/fl}$		vs	vs	vs		PrxCre$^{+/-}$Nox4$^{fl/fl}$		vs	vs	vs
Parameters	Abbreviation	Units	Nox4$^{fl/fl}$	CKO	Nox4$^{-/-}$	CKO	Nox4$^{-/-}$	Nox4$^{-/-}$	Nox4$^{fl/fl}$	CKO	Nox4$^{-/-}$	CKO	Nox4$^{-/-}$	Nox4$^{-/-}$
Tissue volume	TV	mm^3	4.18±0.48	4.09±0.29	4.4±0.38	0.315	0.054	0.203	3.53±0.18	3.58±0.21	3.97±0.34	0.327	0.020	0.010
Bone volume	BV	mm^3	0.44±0.09	0.39±0.03	0.5±0.04	0.079	0.000	0.102	0.26±0.03	0.25±0.03	0.33±0.09	0.194	0.030	0.060
Percent bone volume	BV/TV	%	10.5±1.43	9.64±0.8	11.5±1.12	0.072	0.002	0.104	7.49±0.78	6.94±0.58	8.14±1.58	0.081	0.048	0.188
Tissue surface	TS	mm^2	17.96±1.39	17.59±0.78	18.77±0.39	0.250	0.004	0.116	15.86±0.59	15.83±0.68	17.07±1.02	0.468	0.018	0.016
Bone surface	BS	mm^2	39.25±7.49	35.9±2.6	43.51±3.79	0.113	0.000	0.134	24.89±2.72	23.56±2.43	29.77±6.64	0.176	0.024	0.056
Intersection surface	i.S	mm^2	1.28±0.28	1.14±0.15	1.44±0.22	0.100	0.005	0.157	0.82±0.07	0.78±0.1	0.99±0.32	0.216	0.070	0.100
Bone surface / volume ratio	BS/BV	1/mm	89.21±3.91	91.37±2.16	86.33±3.55	0.086	0.003	0.104	94.26±1.99	94.92±3.39	92.13±4.61	0.332	0.138	0.153
Bone surface density	BS/TV	1/mm	9.32±1.01	8.8±0.61	9.9±0.61	0.105	0.004	0.138	7.05±0.66	6.58±0.37	7.45±1.1	0.061	0.039	0.235
Trabecular pattern factor	Tb.Pf	1/mm	30.77±2.41	32.4±1.53	29.39±2.15	0.056	0.005	0.159	33.18±1.76	33.9±2	32.64±3.89	0.245	0.244	0.376
Centroid (x)	Crd.X	mm	1.28±0.15	1.27±0.13	1.22±0.24	0.439	0.311	0.293	1.16±0.06	1.23±0.09	1.23±0.12	0.058	0.495	0.105
Centroid (y)	Crd.Y	mm	1.57±0.15	1.65±0.1	1.63±0.22	0.115	0.390	0.309	1.46±0.09	1.61±0.16	1.47±0.22	0.028	0.129	0.450
Centroid (z)	Crd.Z	mm	10.23±1.02	10.17±1.24	9.24±2.02	0.459	0.150	0.130	9.94±1.6	9.47±1.35	10.24±1.7	0.283	0.213	0.387
Structure model index	SMI		2.07±0.08	2.13±0.06	2.04±0.07	0.049	0.019	0.278	2.11±0.07	2.14±0.06	2.12±0.15	0.208	0.370	0.447
Trabecular thickness	Tb.Th	mm	0.04±0	0.04±0	0.04±0	0.215	0.005	0.044	0.04±0	0.04±0	0.04±0	0.437	0.192	0.067
Trabecular number	Tb.N	1/mm	2.55±0.3	2.38±0.19	2.71±0.21	0.082	0.005	0.171	1.9±0.2	1.76±0.11	2.03±0.35	0.060	0.043	0.236
Trabecular separation	Tb.Sp	mm	0.21±0.02	0.21±0.01	0.2±0.01	0.298	0.004	0.084	0.26±0.03	0.27±0.02	0.24±0.01	0.217	0.022	0.187
Eigenvalue 1			1.55±0.52	1.59±0.35	1.76±0.41	0.435	0.215	0.236	1.24±0.16	1.32±0.2	1.5±0.11	0.229	0.067	0.011
Eigenvalue 2			3.65±1.31	3.44±0.7	3.85±0.8	0.343	0.172	0.384	2.67±0.28	2.74±0.34	3.19±0.3	0.334	0.027	0.008
Eigenvalue 3			3.86±1.41	3.76±0.71	4.03±0.8	0.426	0.263	0.406	2.88±0.32	3.01±0.36	3.44±0.3	0.253	0.037	0.009
BMD		g/cm3	0.14±0.05	0.13±0.05	0.12±0.03	0.359	0.121	0.318	0.13±0.05	0.14±0.04	0.13±0.02	0.352	0.339	0.491

Supplemental Table 4. Micro-CT trabecular bone parameters of femur of 6-week old male and female Nox4$^{fl/fl}$ control, and PrxCre^{+}Nox4$^{fl/fl}$ CKO and Nox4$^{-/-}$ mice. Animal numbers were indicated in Figure 7 legend, data was expressed as mean ± SD, p value was analyzed by 1-way ANOVA with posthoctests, CKO versus f/f control or Nox4$^{-/-}$.

Supplemental Table 5. **Femur** bone micro-CT cortical bone parameters in 6-week old male and female wild type and PreCre^{+}/-Nox4$^{fl/fl}$ (CKO) and Nox4$^{-/-}$ mice

			Males						Females					
						Nox4$^{fl/fl}$	CKO	Nox4$^{fl/fl}$				Nox4$^{fl/fl}$	CKO	Nox4$^{fl/fl}$
				PrxCre$^{+/-}$Nox4$^{fl/fl}$		vs	vs	vs		PrxCre$^{+/-}$Nox4$^{fl/fl}$		vs	vs	vs
Parameters	Abbreviation	Units	Nox4$^{fl/fl}$	CKO	Nox4$^{-/-}$	CKO	Nox4$^{-/-}$	Nox4$^{-/-}$	Nox4$^{fl/fl}$	CKO	Nox4$^{-/-}$	CKO	Nox4$^{-/-}$	Nox4$^{-/-}$
Tissue volume	TV	mm^3	2.16±0.26	2.07±0.12	2.3±0.1	0.187	0.002	0.136	1.98±0.11	1.91±0.15	2.02±0.17	0.193	0.162	0.319
Bone volume	BV	mm^3	0.8±0.08	0.76±0.02	0.81±0.06	0.082	0.017	0.356	0.71±0.06	0.67±0.05	0.72±0.07	0.123	0.113	0.394
Percent bone volume	BV/TV	%	36.99±1.82	36.7±2.34	35.25±2.25	0.391	0.142	0.077	35.78±1.5	35.29±2.19	35.65±2.25	0.317	0.399	0.456
Tissue surface	TS	mm^2	12.34±1.83	12.87±2.68	11.35±0.37	0.324	0.120	0.132	10.49±0.93	10.32±1.06	11.49±2.37	0.381	0.140	0.167
Peripheral tissue surface	TS(per)	mm^2	6.19±0.6	6.25±0.73	6.05±0.14	0.424	0.281	0.318	5.61±0.19	5.57±0.25	5.98±0.71	0.383	0.091	0.099
Bone surface	BS	mm^2	15.05±1.17	14.49±0.71	15.81±0.94	0.123	0.006	0.125	13.4±0.63	12.93±0.5	13.75±0.95	0.073	0.043	0.235
Peripheral bone surface	BS(per)	mm^2	11.56±0.76	11.19±0.49	12.06±0.56	0.125	0.005	0.116	10.56±0.39	10.28±0.36	10.84±0.64	0.096	0.046	0.193
Bone surface / volume ratio	BS/BV	1/mm	18.93±0.96	19.09±0.63	19.49±1.01	0.346	0.184	0.167	18.98±0.77	19.23±0.74	19.19±0.86	0.272	0.471	0.339
Mean total crossectional tissue area	T.Ar	mm^2	2.14±0.26	2.05±0.12	2.28±0.1	0.187	0.002	0.136	1.96±0.11	1.89±0.15	2±0.16	0.193	0.162	0.319
Mean total crossectional tissue perimeter	T.Pm	mm	6.13±0.6	6.19±0.72	5.99±0.14	0.424	0.281	0.318	5.55±0.19	5.51±0.25	5.92±0.7	0.383	0.091	0.099
Mean total crossectional bone area	B.Ar	mm^2	0.79±0.08	0.75±0.02	0.8±0.06	0.082	0.017	0.356	0.7±0.06	0.67±0.05	0.71±0.07	0.123	0.113	0.394
Mean total crossectional bone perimeter	B.Pm	mm	11.44±0.75	11.08±0.49	11.94±0.55	0.125	0.005	0.116	10.46±0.39	10.18±0.36	10.73±0.63	0.096	0.046	0.193
Average object area per slice	Av.Obj.Ar	mm^2	0.79±0.08	0.75±0.02	0.8±0.06	0.082	0.017	0.356	0.7±0.06	0.67±0.05	0.71±0.07	0.123	0.113	0.394
Average object area-equivalent circle diameter per slice	Av.Obj.ECDa	mm	1±0.05	0.98±0.01	1.01±0.04	0.097	0.017	0.353	0.94±0.04	0.92±0.03	0.95±0.05	0.128	0.118	0.397
Average moment of inertia (x)	Av.MMI(x)	mm^4	0.29±0.04	0.27±0.02	0.32±0.04	0.203	0.008	0.082	0.23±0.03	0.22±0.03	0.26±0.06	0.258	0.066	0.103
Average moment of inertia (y)	Av.MMI(y)	mm^4	0.18±0.04	0.71±0.03	0.18±0.03	0.064	0.017	0.489	0.15±0.02	0.13±0.02	0.15±0.01	0.321	0.123	0.451
Mean polar moment of inertia	MMI(polar)	mm^4	0.47±0.08	0.44±0.03	0.51±0.05	0.106	0.003	0.204	0.37±0.05	0.35±0.05	0.41±0.07	0.175	0.068	0.185
Average principal moment of inertia (max)	Av.MMI(max)	mm^4	0.31±0.05	0.29±0.02	0.35±0.04	0.140	0.003	0.135	0.25±0.03	0.23±0.03	0.28±0.05	0.191	0.055	0.134
Average principal moment of inertia (min)	Av.MMI(min)	mm^4	0.16±0.03	0.14±0.01	0.16±0.02	0.073	0.010	0.412	0.13±0.01	0.12±0.01	0.13±0.02	0.150	0.130	0.367
Mean eccentricity	Ecc		0.71±0.02	0.71±0.03	0.73±0.01	0.221	0.125	0.019	0.7±0.01	0.7±0.02	0.72±0.02	0.467	0.038	0.009
Crossectional thickness	Cs.Th	mm	0.14±0.01	0.14±0	0.13±0.01	0.259	0.379	0.259	0.13±0.01	0.13±0.01	0.13±0.01	0.201	0.356	0.367
Trabecular thickness (plate model)	Tb.Th(pl)	mm	0.11±0.01	0.1±0	0.1±0.01	0.328	0.199	0.174	0.11±0	0.1±0	0.1±0	0.269	0.467	0.341
Trabecular separation (plate model)	Tb.Sp(pl)	mm	0.18±0.02	0.18±0.02	0.19±0.01	0.458	0.191	0.156	0.19±0.01	0.19±0.02	0.19±0.02	0.371	0.401	0.475
Trabecular number (plate model)	Tb.N(pl)	1/mm	3.5±0.24	3.5±0.21	3.43±0.09	0.498	0.239	0.266	3.39±0.06	3.39±0.18	3.42±0.22	0.492	0.408	0.376
Trabecular diameter (rod model)	Tb.Dm(rd)	mm	0.21±0.01	0.21±0.01	0.21±0.01	0.328	0.199	0.174	0.21±0.01	0.21±0.01	0.21±0.01	0.269	0.467	0.341
Trabecular separation (rod model)	Tb.Sp(rd)	mm	0.1±0.01	0.1±0.01	0.1±0.01	0.463	0.194	0.162	0.1±0	0.1±0.01	0.1±0.01	0.380	0.402	0.468
Trabecular number (rod model)	Tb.N(rd)	1/mm	3.25±0.18	3.26±0.11	3.26±0.09	0.431	0.495	0.440	3.2±0.07	3.22±0.11	3.23±0.15	0.353	0.446	0.324
Closed porosity (percent)	Po(cl)	%	61.3±4.84	61.49±4.67	64.69±2.23	0.467	0.090	0.087	64.04±1.5	63.94±3.94	62.85±4.95	0.477	0.347	0.278
Centroid (x)	Crd.X	mm	1.33±0.13	1.29±0.16	1.32±0.19	0.301	0.382	0.459	1.26±0.13	1.35±0.12	1.21±0.07	0.097	0.034	0.283
Centroid (y)	Crd.Y	mm	1.56±0.14	1.64±0.19	1.52±0.1	0.174	0.106	0.288	1.48±0.15	1.51±0.17	1.46±0.12	0.372	0.338	0.441
Centroid (z)	Crd.Z	mm	8.62±1.04	8.53±1.26	7.62±2.01	0.441	0.155	0.128	8.21±1.57	7.7±1.32	8.47±1.7	0.261	0.211	0.402
Mean fractal dimension	FD		1.24±0.02	1.24±0.02	1.25±0.01	0.453	0.167	0.197	1.22±0.02	1.22±0.02	1.22±0.01	0.421	0.341	0.419
Total intersection surface	i.S	mm^2	6.1±0.45	6.08±0.49	6.01±0.15	0.465	0.393	0.351	5.57±0.18	5.55±0.25	5.9±0.56	0.431	0.091	0.089

Supplemental Table 5. Micro-CT cortical bone parameters of femur of 6-week old male and female Nox4$^{fl/fl}$ control, and PrxCre^{+}Nox4$^{fl/fl}$ CKO and Nox4$^{-/-}$ mice. Animal numbers were indicated in Figure 7 legend, data was expressed as mean ± SD, p value was analyzed by 1-way ANOVA with posthoctests, CKO versus f/f control or Nox4$^{-/-}$.

Supplemental Table 6. **Femur** bone micro-CT trabecular bone parameters in 13-week old male and female wild type and PreCre^{+}/-Nox4$^{fl/fl}$ (CKO) and Nox4$^{-/-}$ mice

			Males						Females					
						Nox4$^{fl/fl}$	CKO	Nox4$^{fl/fl}$				Nox4$^{fl/fl}$	CKO	Nox4$^{fl/fl}$
				PrxCre$^{+/-}$Nox4$^{fl/fl}$		vs	vs	vs		PrxCre$^{+/-}$Nox4$^{fl/fl}$		vs	vs	vs
Parameters	Abbreviation	Units	Nox4$^{fl/fl}$	CKO	Nox4$^{-/-}$	CKO	Nox4$^{-/-}$	Nox4$^{-/-}$	Nox4$^{fl/fl}$	CKO	Nox4$^{-/-}$	CKO	Nox4$^{-/-}$	Nox4$^{-/-}$
Tissue volume	TV	mm^3	4.15±0.4	4±0.2	4.06±0.48	0.188	0.384	0.188	3.28±0.28	3.19±0.15	3.2±0.18	0.206	0.450	0.206
Bone volume	BV	mm^3	0.47±0.08	0.45±0.04	0.53±0.17	0.274	0.120	0.274	0.21±0.02	0.22±0.02	0.21±0.03	0.158	0.138	0.158
Percent bone volume	BV/TV	%	11.26±1.71	11.18±0.87	11.93±1.7	0.451	0.162	0.451	6.32±0.58	6.93±0.83	6.43±0.94	0.084	0.132	0.084
Tissue surface	TS	mm^2	18.29±1.05	18.03±0.53	18.34±1.66	0.279	0.324	0.279	15.55±0.82	15.37±0.32	15.62±0.54	0.275	0.130	0.275
Bone surface	BS	mm^2	38.09±5.45	36.4±1.7	41.42±10.02	0.221	0.108	0.221	19.3±1.62	20.01±1.78	19.05±2.25	0.235	0.171	0.235
Intersection surface	i.S	mm^2	1.36±0.29	1.35±0.19	1.63±0.53	0.472	0.104	0.472	0.67±0.17	0.68±0.09	0.69±0.11	0.456	0.437	0.456
Bone surface / volume ratio	BS/BV	1/mm	81.99±5.02	81.73±5.2	80.14±7.2	0.459	0.326	0.459	93.5±3.49	91.01±2.59	93.2±3.74	0.070	0.087	0.070
Bone surface density	BS/TV	1/mm	9.17±0.91	9.1±0.27	10.09±1.23	0.424	0.031	0.424	5.9±0.4	6.29±0.61	5.96±0.65	0.109	0.152	0.109
Trabecular pattern factor	Tb.Pf	1/mm	27.11±3.96	28.5±1.87	26.11±5.2	0.203	0.140	0.203	34.88±2.06	33.18±1.65	35.3±2.22	0.053	0.019	0.053
Centroid (x)	Crd.X	mm	1.28±0.08	1.28±0.1	1.32±0.06	0.483	0.224	0.483	1.23±0.19	1.26±0.11	1.33±0.13	0.385	0.121	0.385
Centroid (y)	Crd.Y	mm	1.5±0.07	1.49±0.1	1.49±0.11	0.375	0.480	0.375	1.38±0.26	1.49±0.13	1.39±0.11	0.145	0.050	0.145
Centroid (z)	Crd.Z	mm	10.52±0.86	10.9±1.49	10.68±1.58	0.256	0.400	0.256	11.03±1.02	10.29±1.78	9.81±0.6	0.206	0.250	0.206
Structure model index	SMI		1.98±0.2	2.09±0.08	1.94±0.24	0.086	0.066	0.086	2.24±0.05	2.19±0.06	2.27±0.05	0.072	0.006	0.072
Trabecular thickness	Tb.Th	mm	0.05±0	0.05±0	0.05±0	0.194	0.365	0.194	0.04±0	0.04±0	0.04±0	0.323	0.397	0.323
Trabecular number	Tb.N	1/mm	2.5±0.33	2.41±0.1	2.78±0.44	0.256	0.026	0.256	1.51±0.11	1.64±0.19	1.51±0.19	0.080	0.094	0.080
Trabecular separation	Tb.Sp	mm	0.2±0.01	0.2±0	0.19±0.01	0.092	0.046	0.092	0.26±0.02	0.25±0.01	0.25±0.01	0.033	0.122	0.033
Eigenvalue 1			2.39±1.02	2.47±0.5	2.44±0.78	0.430	0.471	0.430	1.58±0.19	1.27±0.29	1.63±0.43	0.024	0.027	0.024
Eigenvalue 2			3.87±1.62	3.8±0.78	3.81±1.31	0.459	0.489	0.459	2.33±0.34	1.91±0.37	2.27±0.48	0.026	0.048	0.026
Eigenvalue 3			4.35±1.77	4.33±0.83	4.21±1.53	0.492	0.430	0.492	2.59±0.35	2.1±0.43	2.49±0.54	0.024	0.063	0.024
BMD		g/cm3	0.23±0.03	0.22±0.05	0.26±0.04	0.452	0.107	0.452	0.26±0.09	0.23±0.05	0.21±0.05	0.268	0.192	0.268

Supplemental Table 6. Micro-CT trabecular bone parameters of femur of 13-week old male and female Nox4$^{fl/fl}$ control, and PrxCre^{+}Nox4$^{fl/fl}$ CKO and Nox4$^{-/-}$ mice. Animal numbers were indicated in Figure 7 legend, data was expressed as mean ± SD, p value was analyzed by 1-way ANOVA with posthoctests, CKO versus f/f control or Nox4$^{-/-}$.

Supplemental Table 7. **Femur** bone micro-CT cortical bone parameters in 13-week old male and female wild type and PreCre^{+}/-Nox4$^{fl/fl}$ (CKO) and Nox4$^{-/-}$ mice

			Males						Females					
						Nox4$^{fl/fl}$	CKO	Nox4$^{fl/fl}$				Nox4$^{fl/fl}$	CKO	Nox4$^{fl/fl}$
				PrxCre$^{+/-}$Nox4$^{fl/fl}$		vs	vs	vs		PrxCre$^{+/-}$Nox4$^{fl/fl}$		vs	vs	vs
Parameters	Abbreviation	Units	Nox4$^{fl/fl}$	CKO	Nox4$^{-/-}$	CKO	Nox4$^{-/-}$	Nox4$^{-/-}$	Nox4$^{fl/fl}$	CKO	Nox4$^{-/-}$	CKO	Nox4$^{-/-}$	Nox4$^{-/-}$
Tissue volume	TV	mm^3	2.35±0.2	2.25±0.13	2.29±0.32	0.134	0.387	0.325	1.97±0.14	1.88±0.08	1.9±0.12	0.068	0.288	0.215
Bone volume	BV	mm^3	0.95±0.09	0.94±0.07	0.96±0.13	0.442	0.388	0.421	0.92±0.07	0.89±0.03	0.9±0.04	0.156	0.267	0.324
Percent bone volume	BV/TV	%	40.45±1.85	41.94±1.52	42±1.37	0.050	0.468	0.049	46.66±1.7	47.5±1.62	47.37±1.06	0.186	0.430	0.196
Tissue surface	TS	mm^2	11.92±1.1	11.73±1.28	12.56±2.18	0.377	0.208	0.222	10.71±0.94	9.83±0.28	10.1±0.6	0.007	0.107	0.100
Peripheral tissue surface	TS(per)	mm^2	6.28±0.39	6.18±0.3	6.3±0.44	0.276	0.289	0.477	5.73±0.23	5.49±0.1	5.64±0.31	0.006	0.077	0.306
Bone surface	BS	mm^2	17.87±2.22	17.64±2.04	18.13±3.23	0.416	0.374	0.427	14.45±0.54	14.06±0.38	13.73±0.95	0.064	0.165	0.080
Peripheral bone surface	BS(per)	mm^2	13.15±1.4	12.97±1.42	13.16±2	0.397	0.421	0.495	10.67±0.38	10.41±0.19	10.28±0.62	0.048	0.269	0.122
Bone surface / volume ratio	BS/BV	1/mm	18.8±1.16	18.66±0.86	18.8±0.75	0.392	0.382	0.495	15.81±0.83	15.79±0.46	15.22±0.51	0.480	0.015	0.079
Mean total crossectional tissue area	T.Ar	mm^2	2.33±0.2	2.23±0.13	2.27±0.32	0.134	0.387	0.325	1.95±0.14	1.86±0.08	1.89±0.12	0.068	0.288	0.215
Mean total crossectional tissue perimeter	T.Pm	mm	6.22±0.38	6.12±0.29	6.23±0.44	0.276	0.289	0.477	5.67±0.23	5.43±0.1	5.59±0.31	0.006	0.077	0.306
Mean total crossectional bone area	B.Ar	mm^2	0.94±0.08	0.93±0.07	0.95±0.13	0.442	0.388	0.421	0.91±0.07	0.88±0.03	0.89±0.04	0.156	0.267	0.324
Mean total crossectional bone perimeter	B.Pm	mm	13.02±1.39	12.84±1.4	13.03±1.98	0.397	0.421	0.495	10.57±0.38	10.31±0.19	10.18±0.62	0.048	0.269	0.122
Average object area per slice	Av.Obj.Ar	mm^2	0.94±0.08	0.93±0.07	0.95±0.13	0.442	0.388	0.421	0.91±0.07	0.88±0.03	0.89±0.04	0.156	0.267	0.324
Average object area-equivalent circle diameter per slice	Av.Obj.ECDa	mm	1.09±0.05	1.09±0.04	1.1±0.08	0.449	0.406	0.436	1.07±0.04	1.06±0.02	1.07±0.03	0.164	0.275	0.329
Average moment of inertia (x)	Av.MMI(x)	mm^4	0.42±0.08	0.41±0.08	0.43±0.14	0.413	0.391	0.442	0.3±0.05	0.28±0.02	0.3±0.04	0.165	0.187	0.418
Average moment of inertia (y)	Av.MMI(y)	mm^4	0.19±0.03	0.18±0.02	0.19±0.05	0.119	0.290	0.416	0.17±0.02	0.15±0.02	0.16±0.03	0.062	0.206	0.269
Mean polar moment of inertia	MMI(polar)	mm^4	0.62±0.11	0.59±0.09	0.62±0.17	0.317	0.359	0.478	0.47±0.07	0.43±0.03	0.45±0.05	0.087	0.129	0.344
Average principal moment of inertia (max)	Av.MMI(max)	mm^4	0.45±0.08	0.44±0.08	0.46±0.13	0.426	0.410	0.454	0.33±0.06	0.31±0.02	0.33±0.04	0.118	0.079	0.435
Average principal moment of inertia (min)	Av.MMI(min)	mm^4	0.17±0.02	0.15±0.01	0.16±0.04	0.064	0.213	0.444	0.14±0.02	0.12±0.01	0.13±0.02	0.047	0.360	0.145
Mean eccentricity	Ecc		0.79±0.02	0.81±0.03	0.8±0.02	0.086	0.257	0.288	0.76±0.02	0.77±0.02	0.78±0.02	0.212	0.092	0.047
Crossectional thickness	Cs.Th	mm	0.14±0.01	0.15±0.01	0.15±0	0.361	0.475	0.338	0.17±0.01	0.17±0	0.18±0.01	0.439	0.044	0.195
Trabecular thickness (plate model)	Tb.Th(pl)	mm	0.11±0.01	0.11±0	0.11±0	0.408	0.373	0.476	0.13±0.01	0.13±0	0.13±0	0.494	0.014	0.079
Trabecular separation (plate model)	Tb.Sp(pl)	mm	0.16±0.01	0.15±0.01	0.15±0.01	0.098	0.387	0.037	0.15±0.01	0.14±0.01	0.15±0	0.214	0.105	0.398
Trabecular number (plate model)	Tb.N(pl)	1/mm	3.8±0.21	3.91±0.27	3.95±0.16	0.165	0.405	0.078	3.69±0.17	3.75±0.18	3.6±0.09	0.259	0.035	0.155
Trabecular diameter (rod model)	Tb.Dm(rd)	mm	0.21±0.01	0.21±0.01	0.21±0.01	0.408	0.373	0.476	0.25±0.01	0.25±0.01	0.26±0.01	0.494	0.014	0.079
Trabecular separation (rod model)	Tb.Sp(rd)	mm	0.08±0.01	0.08±0.01	0.08±0	0.094	0.390	0.036	0.08±0.01	0.07±0.01	0.08±0	0.208	0.131	0.466
Trabecular number (rod model)	Tb.N(rd)	1/mm	3.37±0.18	3.41±0.19	3.44±0.13	0.340	0.390	0.228	3.04±0.14	3.07±0.11	2.95±0.08	0.356	0.017	0.095
Mean trabecular pattern factor	Tb.Pf	1/mm	-1.12±0.51	-1.14±0.6	-1.32±0.62	0.468	0.303	0.246	-0.76±0.19	-0.71±0.21	-0.59±0.28	0.313	0.160	0.125
Closed porosity (percent)	Po(cl)	%	59.38±1.79	57.87±1.46	57.5±1.79	0.043	0.345	0.031	53.11±1.66	52.39±1.64	52.48±1.02	0.220	0.451	0.216
Centroid (x)	Crd.X	mm	1.27±0.11	1.21±0.11	1.23±0.17	0.149	0.396	0.299	1.23±0.2	1.17±0.14	1.28±0.14	0.248	0.056	0.296
Centroid (y)	Crd.Y	mm	1.66±0.17	1.57±0.16	1.49±0.08	0.146	0.132	0.017	1.4±0.09	1.41±0.08	1.37±0.09	0.420	0.163	0.276
Centroid (z)	Crd.Z	mm	9.15±0.88	9.53±1.48	9.28±1.58	0.259	0.388	0.417	9.6±1.05	8.83±1.78	8.35±0.61	0.198	0.252	0.013
Mean fractal dimension	FD		1.28±0.03	1.29±0.02	1.3±0.05	0.337	0.288	0.189	1.26±0.02	1.26±0.03	1.25±0.02	0.412	0.135	0.193
Total intersection surface	i.S	mm^2	6.24±0.3	6.16±0.29	6.25±0.44	0.311	0.345	0.478	5.68±0.24	5.48±0.1	5.58±0.21	0.018	0.104	0.228

Supplemental Table 7. Micro-CT cortical bone parameters of femur of 6-week old male and female Nox4$^{fl/fl}$ control, and PrxCre^{+}Nox4$^{fl/fl}$ CKO and Nox4$^{-/-}$ mice. Animal numbers were indicated in Figure 7 legend, data was expressed as mean ± SD, p value was analyzed by 1-way ANOVA with posthoc tests, CKO versus f/f control or Nox4$^{-/-}$.

Supplementary Table 8. Chemical reagents, antibodies, proteins, cell lines, mice and assay kits used for this investigation

**Chemical Reagents**	**Source**	**Catalogue #**
Amido Black Staining Solution	Sigma (MO, USA)	A8181
MEM α, nucleosides, no phenol red	ThermoFisher (MA, USA)	41061029
Fetal Bovine Serum (FBS), United States	ThermoFisher (MA, USA)	26140079
Penicillin-Streptomycin (5,000 U/mL)	ThermoFisher (MA, USA)	15070063
Recombinant Mouse TRANCE/TNFSF11/RANK L	R&D Systems (MN, USA)	462-TEC
Acid Phosphatase, Leukocyte Kit	Sigma (MO, USA)	386
Osteo Assay Surface Microplate	Corning (AZ, USA)	CLS3989
Formalin, Buffered PBS, 10%	FisherScientific (MA, USA)	SF100-4
Glutaraldehyde 2.5% in Phosphate Buffer	FisherScientific (MA, USA)	50-366-996
Von Kossa Stain Kit	Abcam (MA, USA)	ab150687
TRI Reagent™ Solution	Invitrogen (CA, USA)	AM9738
DNase Max Kit	QIAGEN (MD, USA)	15200-50
iScript™ cDNA Synthesis Kit	Bio-Rad (CA, USA)	1708890
Fast SYBR™ Green Master Mix	ThermoFisher (MA, USA)	4385612
RIPA Lysis and Extraction Buffer	ThermoFisher (MA, USA)	89901
SuperSignal™ West Pico PLUS Chemiluminescent Substrate	ThermoFisher (MA, USA)	34577
DNeasy PowerSoil Pro Kit	QIAGEN (MD, USA)	47014
**Antibodies**	**Source**	**Catalogue #**
Prx1	OriGene	Cat# AP23064PU-N
CathepsinK	Abcam (MA, USA)	ab187647
**Assay Kits**	**Source**	**Catalogue #**
Cell-based ROS/superoxide detection assay kit	Abcam (abcam.com)	cat#ab139476
Non-Radioactive cell proliferation assay	Promega Corporation	Part#TB169
CTX 1	MyBioSource (CA, USA)	MBS722404
P1NP	MyBioSource (CA, USA)	MBS2500076
Cathepsin K	mybiosource.com	cat#MBS164601
**Mice**	**Source**	**Catalogue #**
Nox4$^{fl/fl}$	Louisiana State University Health Sciences Center, New Orleans	
PrxCre$^{+/-}$ *Nox4*$^{fl/fl}$	Louisiana State University Health Sciences Center, New Orleans	

Nox4$^{-/-}$	Louisiana State University Health Sciences Center, New Orleans	
**Animal Diet**	**Source**	**Catalogue #**
AIN-93G diet	Harlan Industries, Indianapolis, IN	

Supplementary Table 9. Sequence of primer.

Gene	Sequence (5' – 3')	
	Forward	Reverse
mNox1	ATG CCC CTG CTG CTC GAA TA	AAA TTG CCC CTC CAT TTC CT
mGAPDH	GTA TGA CTC CAC TCA CGG CAA A	GGT CTC GCT CCT GGA AGA TG
mTNFa	GAC GTG GAA CTG GCA GAA GAG	GCC ACA AGC AGG AAT GAG AAG
mTRAP	TGG TCC AGG AGC TTA ACT GC	GTC AGG AGT GGG AGC CAT ATG
mCathepsinK	GTG GGT GTT CAA GTT TCT GC	GGT GAG TCT TCT TCC ATA GC
mNox2	ACC GCC ATC CAC ACA ATT G	CCG ATG TCA GAG AGA GCT ATT GAA
mNox4	CTG CAT CTG TCC TGA ACC TCA A	TCT CCT GCT AGG GAC CTT CTG T
mALP	TAA CCG CTA CCC GGA TCC TA	TGT CTT GGA CAG AGC CAT GTG
mb-Catenin	AAC TGC GTG GAT GGG ATC TG	GAT ATT GAC GGG CAG TAT GCA A
mNFaTc1	GATCCCGTTGCTTCCAGAAAAT	TCTGTCTCCCCTTTCCTCAGCT
mNFkB	CCT CTGGCGAATGGCTTTAC	GCTATGGAT ACTGCGGTCTGG

Supplementary Results: bone marrow RNA sequencing

Groups:
3 weeks old (3 wk) male (M) and female (F) mice of genotypes *Nox4^fl/fl^* (Flx), *PrxCre*$^{+/-}$ *Nox4*$^{fl/fl}$ (Prx) and *Nox4*$^{-/-}$ (Nox4 0).
32 weeks old (32 wk) male (M) and female (F) mice of genotypes *Nox4*$^{fl/fl}$ (Flx), *PrxCre*$^{+/-}$ *Nox4*$^{fl/fl}$ (Prx) and *Nox4*$^{-/-}$ (Nox4 0).

There were 3 samples per group, with each sample a pool of RNA from 2-4 mice. The data set is thus from a complete 2 x 2 x 3 factorial design with the two sexes, two ages (3 weeks and 32 weeks) and three genotypes (*Nox4*$^{fl/fl}$, *PrxCre*$^{+/}$- *Nox4*$^{fl/fl}$, and *Nox4*$^{-/-}$).

The data set quantified expression for 24623 genes. Of these, 20602 had at least 1 raw count. 14338 genes had an average of at least 10 raw counts per sample.

The 10 genes with the highest average normalized expression were:

Hba-a1	Hemoglobin
Hba-a2	Hemoglobin
Hbb-b1	Hemoglobin
Hbb-bs	Hemoglobin
S100a9	Immune/inflammatory role
Slc4a1	Red blod cell chloride/bicarbonate exchanger
Eef1a1	Translation elongation factor
Ngp	Neutrophilic granule protein
Hbb-b2	Hemoglobin
Hbb-bt	Hemoglobin

These are genes that are expected to be highly expressed in bone marrow.

In terms of abundance, Nox4 was poorly expressed at no. 17601 on the list with very low raw counts:

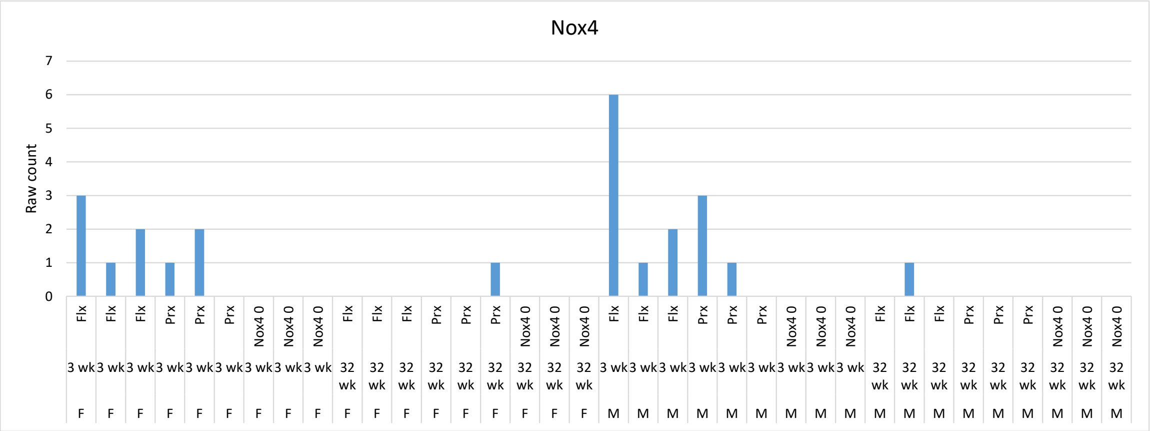

Based on the normalized gene counts, an analysis of variance (ANOVA) for each gene was calculated. We consider ANOVA well-suited for analysis of normalized counts as long as the counts are not too low. An average raw count of at least 10 was chosen as criterion for conducting ANOVA. There were 14338 genes fulfilling this criterion.

The ***main effects*** of Age, Sex and Genotype were explored. They showed least significant difference (LSD), i.e., without adjustments for multiplicity of genes, at $P < 0.05$ for 11711, 2784 and 3533 genes, respectively. These numbers were higher than what one would expect by chance (14338 * 0.05 = 717 genes).

The following figure shows partitioning of the variance for genes for which there was a significant treatment effect at LSD $P < 0.05$.

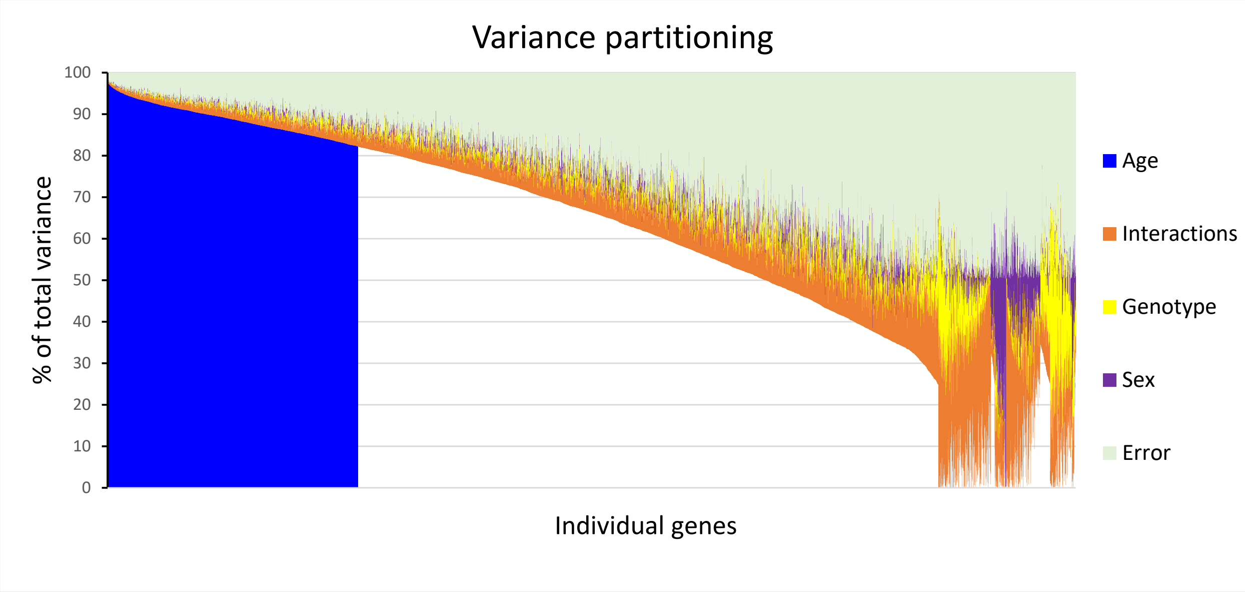

It underscores that for most genes, age is the most important variable affecting gene expression.

Age

The expression of most genes was highly dependent on the age of the mice.

The gene with lowest P-value for a main effect of age was Efemp1, which essentially was only expressed in the older mice.

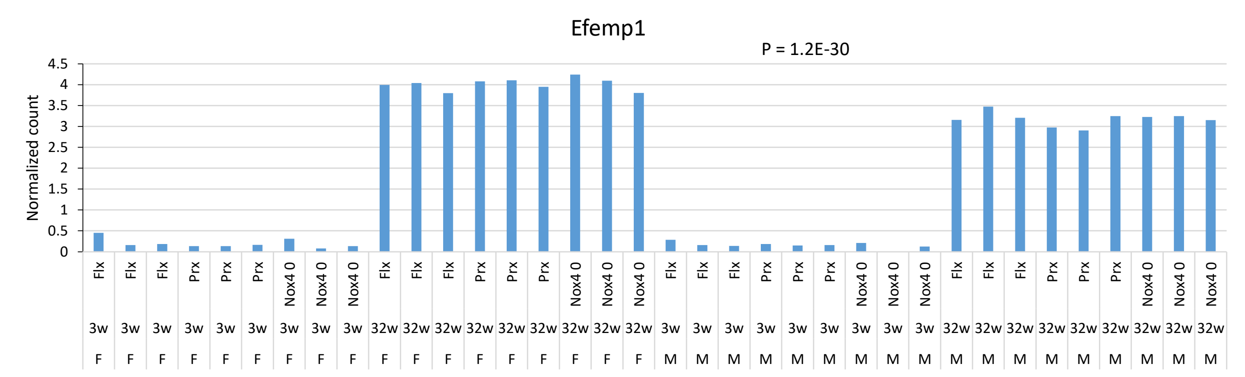

Volcano plot for main effect of age:

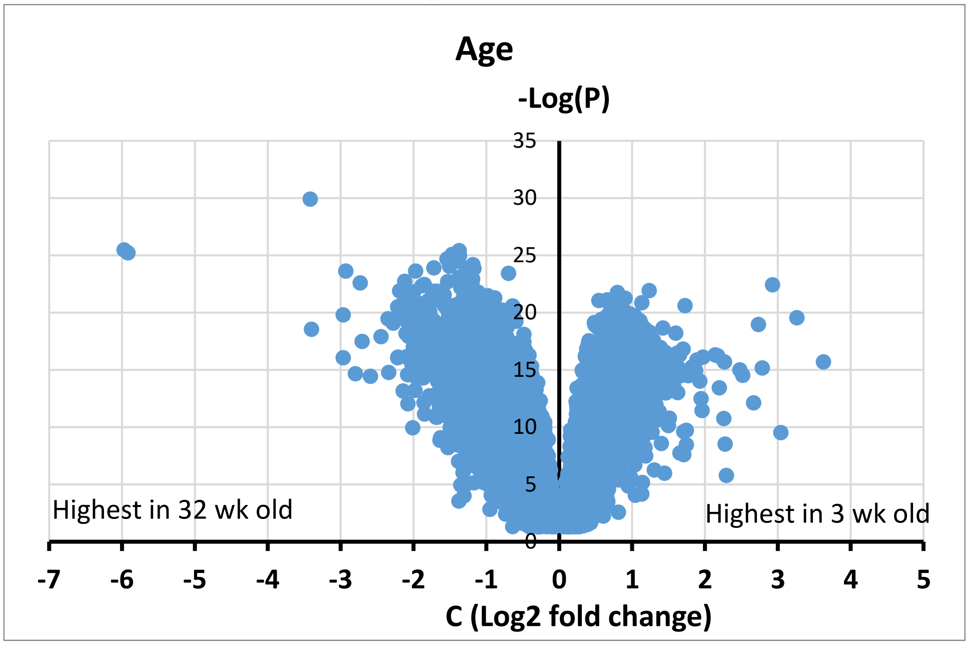

Many genes had > 2-fold expression difference (absolute value of C > 1) at 3 and 32 weeks of age. The following heat map are for genes with a main significant age difference and an absolute value of C > 1:

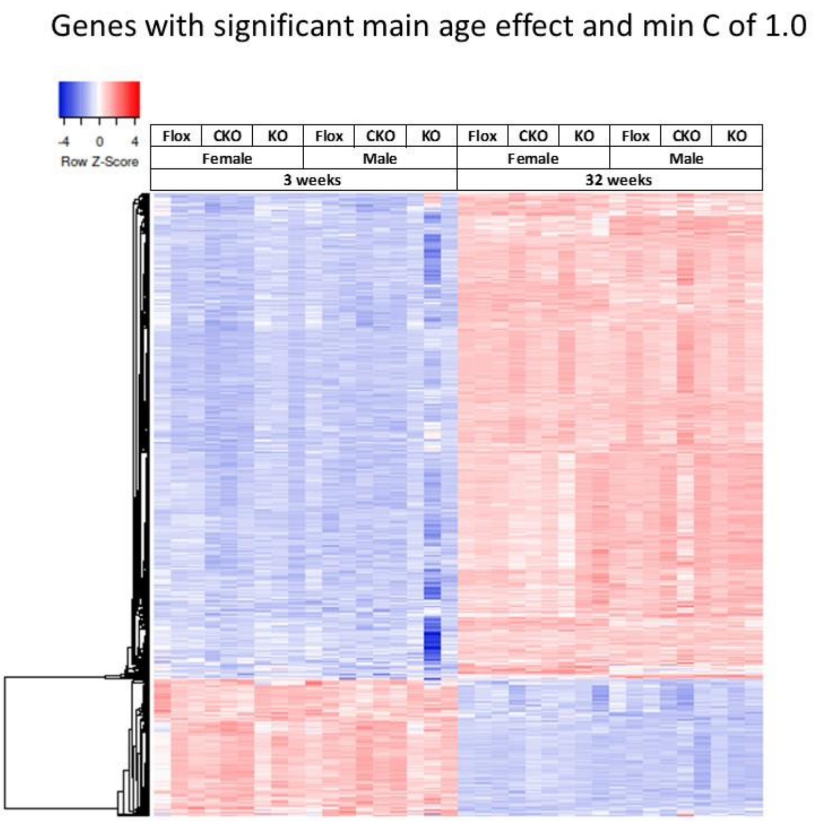

<u>There were genes with highest expression at either 3 weeks of age or 32 weeks of age with the latter groups being most abundant.</u>

<u>Sex</u>
Volcano plot for main effect of sex:

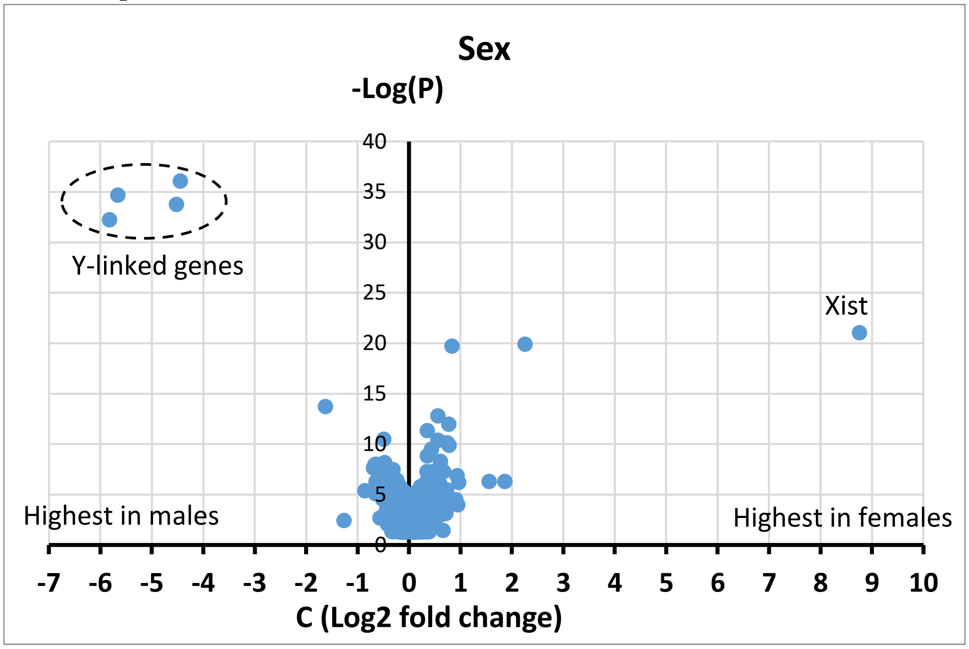

The 4 genes with male-exclusive expression are the Y-linked genes *Kdm5d*, *Eif2s3y*, *Uty* and *Ddx*3y.

Xist is an X-linked gene responsible for X-chromosome inactivation in females.

The following heat map are for genes with a main significant sex difference and an absolute value of C > 0.3.

Genes with significant main sex effect and min C of 0.3

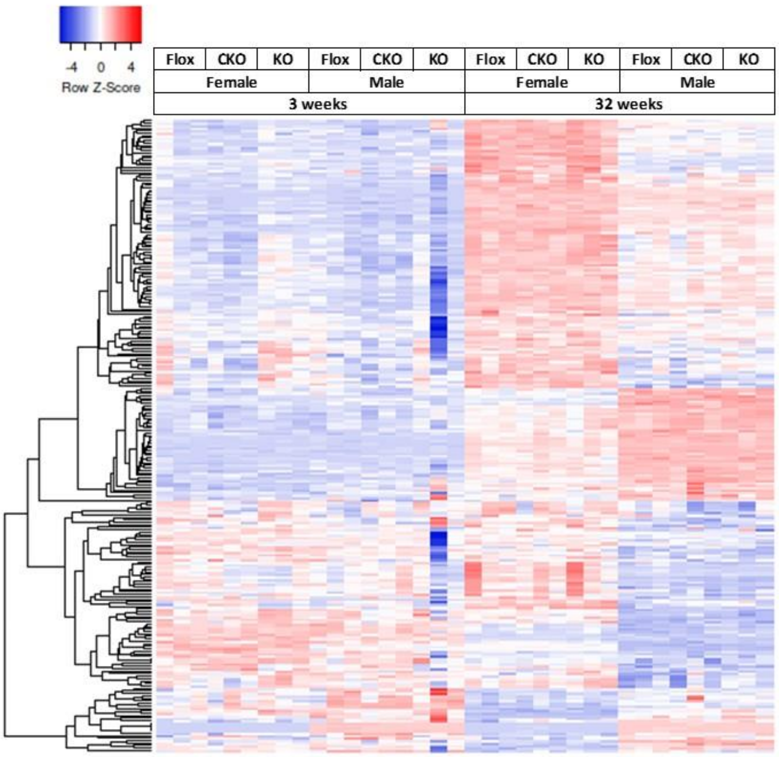

The sex effect was most pronounced at 32 weeks of age.

Genotype

The gene with the strongest genotype effect was *Cyp2s1*.

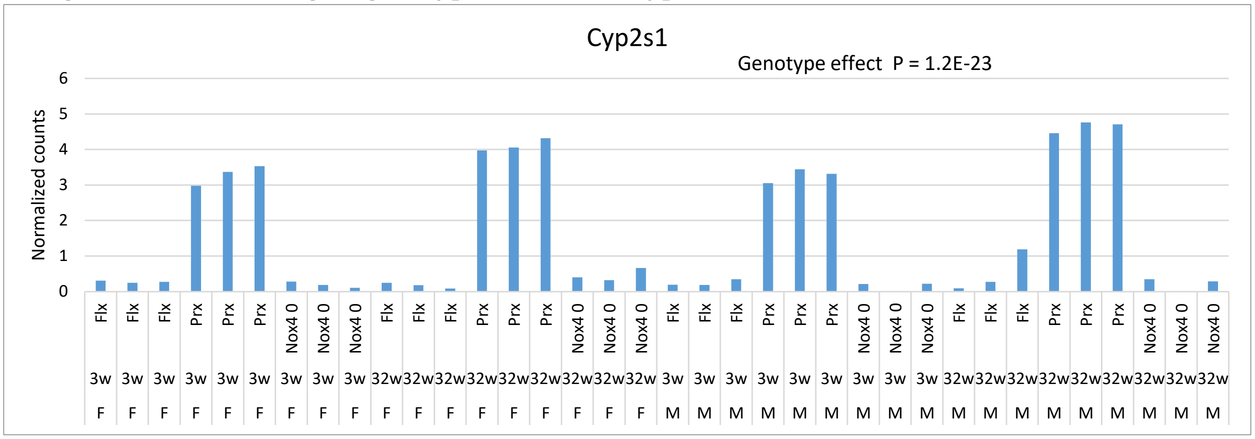

It was expressed predominantly in the *PrxCre +/- Nox4 fl/fl* genotype.

The main genotype effect with two degrees of freedom was separated into two orthogonal contrasts. The first was a contrast of the expression in the conditional knockout versus the expression in the whole body knockout. The second compared the expression in the control *Nox4 fl/fl* genotype with the average expression of the two knockout genotypes. These two contrasts are significant at LSD P < 0.05 for 4275 and 749 genes, respectively.

Volcano plots:

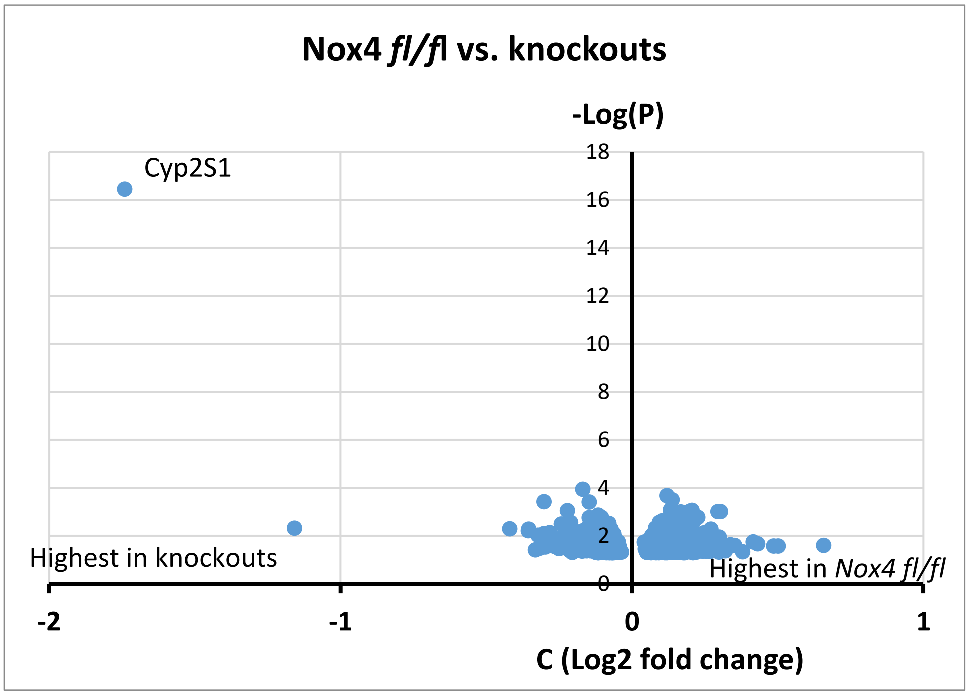

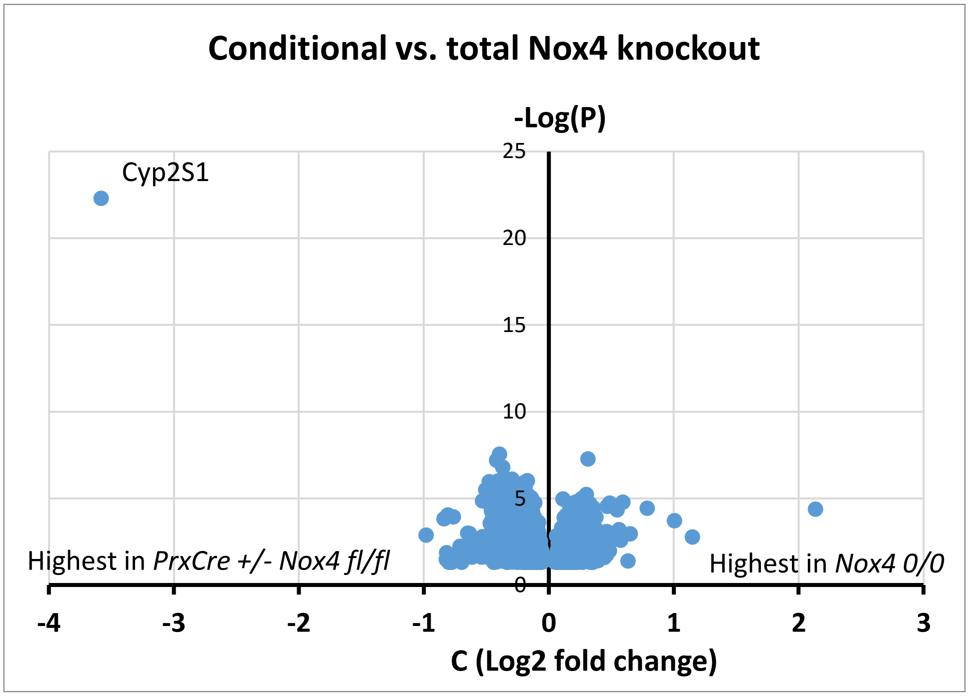

The following heatmap are for genes showing a significant (LSD P < 0.05) difference in pairwise comparisons of any of the three genotypes with an absolute value of C > 0.3:

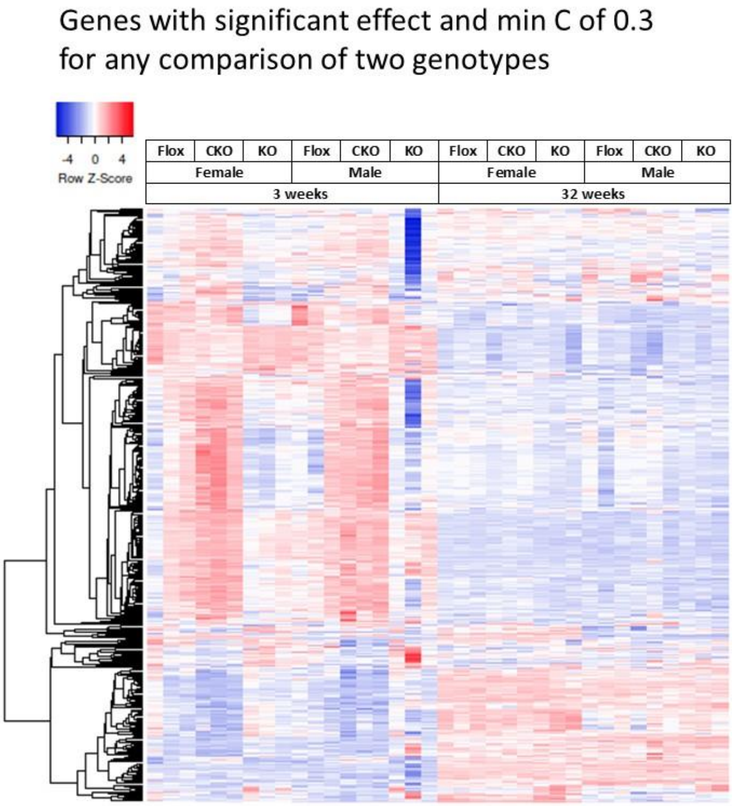

Genotype differences were most apparent at 3 weeks of age with expression in the conditional knockout genotype differing from expression in both the control floxed genotype and the whole body knockout genotype.

It should be noted that the analysis above included one sample of RNA pooled from male Nox4$^{-/-}$ mice at 3 weeks of age resulting in only 406,684 total reads compared to the other samples with a minimum of 11,222,165 total reads. We further explored the data set when the sample with low total reads were eliminated.

A principal components analysis (PCA) is shown below:

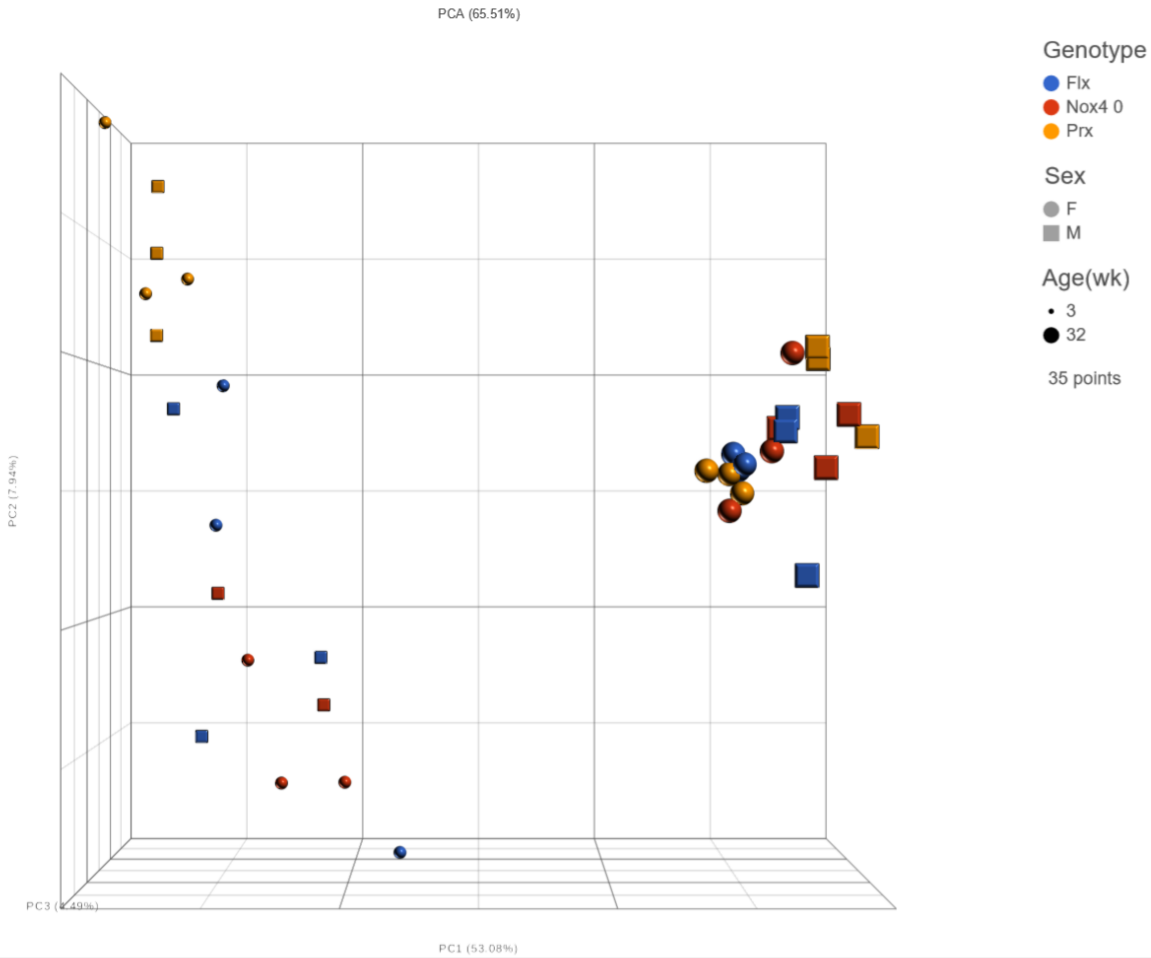

The first principal component separates all samples at 3 weeks of age from all samples at 32 weeks of age, underscoring that age is the most important variable in terms of sample distribution.

The second principal component appears to separate mice of the PrxCre+/- Nox4fl/fl genotype from the other two genotypes at 3 weeks of age.

Thus, the PCA analysis excluding the low count sample essentially shows the same general pattern as the ANOVA analyses including the low count sample.

Gene ontology enrichment analysis was conducted for the analysis of the top 500 most significant genes, i..e. with lowest P-values in the ANOVA analyses, for the main effects of age, sex and genotype. The analysis was conducted using the web-based Gene Ontology Resource (The Gene Ontology Consortium. 2019. The Gene Ontology Resource: 20 years and still GOing strong. Nucleic Acids Research 47:D330-D338).

The gene list for age was enriched in genes involved in e.g. blood coagulation, platelet biology, immune response, osteoblast differentiation and bone development.

The gene list for sex was enriched in genes involved in e.g. collagen fibril organization, extracellular matrix, cartilage development, immune response and the vasculature.

The gene list for genotype was enriched in genes involved in e.g. ribosome assembly, translation, mitochondrial biology and oxidative phosphorylation.
